# *Lobesia botrana* Infestation in Petit Verdot and Sangiovese: A Comparative Study

**DOI:** 10.3390/insects16020213

**Published:** 2025-02-15

**Authors:** Lorenzo Corsi, Giorgio Sperandio, Sara Ruschioni, Fabio Ramilli, Tania Lattanzi, Oriana Silvestroni, Paola Riolo

**Affiliations:** 1 Department of Agricultural, Food and Environmental Sciences, Polytechnic University of Marche, Via Brecce Bianche, 60131 Ancona, Italy; l.corsi@staff.univpm.it (L.C.); g.sperandio@staff.univpm.it (G.S.); t.lattanzi@staff.univpm.it (T.L.); o.silvestroni@staff.univpm.it (O.S.); p.riolo@staff.univpm.it (P.R.); 2 Department of Industrial Engineering, University of Bologna, Viale Risorgimento 4, 40136 Bologna, Italy; fabio.ramilli2@unibo.it

**Keywords:** *Lobesia botrana*, Petit Verdot, Sangiovese, pest impacts, grapevine cultivars, integrated pest management, bunch morphology

## Abstract

The European grapevine moth (*Lobesia botrana*), a significant pest in viticulture, impacts grape quality and yield through larval feeding and secondary infections. This study examined the impact of *L. botrana* on two grape varieties, Petit Verdot and Sangiovese, while also investigating cultivar-specific traits. Field trials were conducted in central Italy to assess infestation levels, female population abundance, and cultivar-specific morphological traits. The results revealed significantly lower larval infestation levels, but higher female abundance in Petit Verdot compared to Sangiovese. Bunch density and bunch compactness were also significantly lower in Petit Verdot than in Sangiovese. Morphological traits, such as a greater canopy thickness and higher leaf layer number in Petit Verdot, did not correspond to increased infestation levels, challenging existing findings regarding the impact of sun exposure on female oviposition and larval settlement. The seasonal dynamics of *L. botrana* indicated three major adult flight peaks and a partial fourth peak, potentially influenced by local weather patterns. These findings highlight the critical role of cultivar-specific traits in shaping pest infestation dynamics and provide essential insights for optimizing integrated pest management (IPM) strategies, particularly in cultivar selection and monitoring protocols for sustainable viticulture.

## Simple Summary

The European grapevine moth (*Lobesia botrana*) is a major pest that reduces grape quality and reduces yields by feeding on berries and promoting secondary infections. This study investigated the impact of *L. botrana* on two grape varieties, Petit Verdot and Sangiovese, and explored cultivar-specific traits in central Italy. Results showed that Petit Verdot suffered less damage than Sangiovese, likely due to the looser structure of its grape bunches, despite capturing more adult moths near these plants. Bunch density and bunch compactness were significantly lower in Petit Verdot compared to Sangiovese. Thus, the denser canopies and thicker foliage found in Petit Verdot did not correspond to increased pest damage, challenging previous assumptions about the role of sun exposure. Seasonal moth activity revealed three main population peaks and a smaller fourth peak, likely influenced by weather conditions. These findings highlight the role of the grape variety in pest damage susceptibility and provide valuable insights for more sustainable pest management strategies, supporting environmentally friendly grape production.

## 1. Introduction

Grapevine (*Vitis vinifera* L. *spp. sativa*) is one of the most important crops worldwide [1,2]. Unfortunately, its economic value is constantly threatened by numerous arthropod pests [3]. Among these, the European grapevine moth, *Lobesia botrana* (Denis & Schiffermüller) (Lepidoptera: Tortricidae), is considered one of the major pests [4,5,6]. This pest is widespread and endemic across all wine-growing areas of the Palearctic Region [7,8,9], with its greatest economic impact in southern Europe [8,10]. In 2009, *L. botrana* was also detected in key wine-producing regions of North and South America [11,12]. However, following an area-wide eradication campaign, the pest was officially declared eradicated in all previously infested areas of California in August 2016 [13]. Depending on the latitude and climatic and microclimatic conditions, *L. botrana* completes between two and five generations per year [8]. In Central and Northern Europe (e.g., Germany, Switzerland, Austria, and northern France), two generations per year are typically observed, whereas in southern Europe (e.g., Italy, southern France, Spain, Portugal, and Greece), three to four generations are more common [14,15,16]. The first larval generation is anthophagous, developing on inflorescences and forming the so-called nests or glomeruli, while the other generations are carpophagous. Direct damage results from larvae tunneling into and feeding on grape berries. Indirect damage arises from bunch rots in injured berries, primarily caused by *Botrytis cinerea* [8,16]. *L. botrana* larvae feed on nearly all grape varieties [17,18,19,20]. However, some cultivars experience greater damage, exhibiting higher infestation levels than others [18,21]. Several studies have shown that oviposition efficiency, larval fitness, and infestation rate vary among cultivars [20,21,22,23,24]. Differences in infestation levels appear to be primarily influenced by cultivar-specific traits, including leaf coverage and bunch compactness. These factors, in turn, modify the microclimatic conditions around and within grape bunches [25,26].

Variability in microclimatic parameters, influenced by cultivar characteristics and canopy management, can significantly impact the reproductive success of *L. botrana* as well as egg and larval viability. Temperature and relative humidity are the primary factors affecting egg and larval survival. For instance, field trials have shown that temperatures above 40 °C, combined with low relative humidity, result in high egg mortality [27,28,29]. Berries exposed to direct sunlight can reach temperatures approximately 10 °C higher than the surrounding air temperature [30,31,32]. As a result, the temperature of berries may exceed the critical survival threshold for *L. botrana* even when air temperature is below this level. Additionally, females have been observed to prefer laying eggs on the side of bunches exposed to sunlight in the hours before oviposition, showing a slight preference for these over leaf-covered bunches [26].

Information and data on the characteristics and the infestation levels of *L. botrana* are available for widespread grapevine cultivars such as Sangiovese, which is notably susceptible to this pest [33,34,35,36,37]. This cultivar of Italian origin is grown on approximately 73,000 hectares (ranking 11th globally), with 93% of its cultivation concentrated in Italy [38]. In contrast, less widespread cultivars remain largely understudied in terms of infestation levels and their relationships with morphological traits such as vigor, canopy density, and bunch compactness. Among these, Petit Verdot, a cultivar of French origin, is gaining global interest. Its acreage expanded from 1640 hectares in 2000 to 8124 hectares in 2016 [38] and it is currently cultivated in the warmer regions of Spain (1804 ha), the United States (1219 ha), Australia (1118 ha), and France (870 ha). In Italy, Petit Verdot covered an estimated 300 hectares in 2016, with a growing trend in recent years due to the high quality of its wines and its positive response to elevated temperatures [38,39].

The aims of this study were (i) to investigate the incidence (Infestation Index—II) and severity (Severity Index—SI) of *L. botrana* infestation on Petit Verdot and Sangiovese cultivars, (ii) to analyze male flight dynamics and abundance on the two cultivars, and (iii) to characterize the morphological traits (shoot, canopy, and bunch traits) of both cultivars.

## 2. Materials and Methods

### 2.1. Experimental Vineyard and Weather Data

This study was conducted in a 3.5 ha vineyard located in central Italy (Loreto, Ancona province, 43°25′47″ N 13°37′14″ E, 75–84 m a.s.l.) during the years 2021 and 2022. The vineyard, established in 1980, consists of Petit Verdot and Sangiovese cultivars on Kober 5BB rootstocks, with row spacing of 3.0 m and vine spacing of 1.3 m along rows. Winter hand pruning left a cane with 10–14 nodes per vine. The cane was set on a supporting wire at 0.8 m aboveground with three pairs of catch wires providing a trellis extending 1.0 m above the cane. Shoots were vertically positioned between the catch wires and mechanically trimmed at the end of June once they exceeded the top wires. The vineyard was sprayed to control powdery mildew and downy mildew, but no insecticide treatments were administered. The surrounding vegetation was homogeneous to minimize potential effects from other host plants. Temperature and relative humidity data were obtained from the regional hydro-meteorological and rainfall monitoring network, managed by the Civil Protection Department of the Marche Region (http://app.protezionecivile.marche.it/sol/indexjs.sol?lang=it (accessed on 15 January 2022)).

### 2.2. Experimental Design

**Figure 1:**
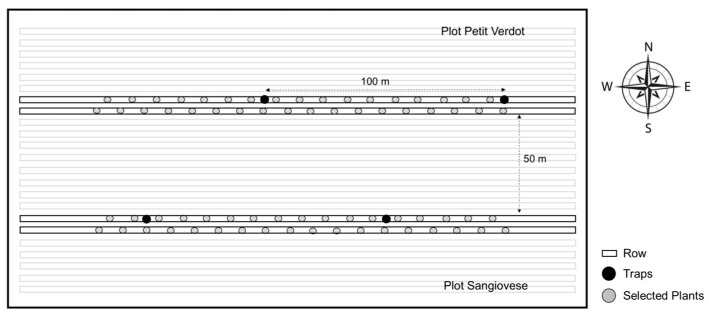
Schematic layout of the experimental field in Loreto, Ancona province (Italy).

In the experimental vineyard, two adjacent rows from the middle of each tested cultivar (Petit Verdot and Sangiovese) were selected, ensuring a minimum distance of 50 m between rows of different cultivars (Figure 1).

In the two selected rows of each cultivar, the *L. botrana* infestation level was assessed through an on-site visual inspection of 35 plants. Each plant represented a replicate. For each monitored plant, all inflorescences and bunches were examined for live larvae, larval nests, or larval penetration holes. To monitor *L. botrana* population abundance, two sex pheromone traps were deployed per cultivar. Shoot and bunch morphology was evaluated on 10 plants per cultivar. Canopy traits were evaluated on four plants per cultivar.

### 2.3. Infestation Indices

Surveys of *L. botrana* larval infestation levels were conducted on 16 June (after the end of the first flight period), 27 July (after the end of the second flight period), and 8 September (a few days before harvest) 2021. In June, all inflorescences of 35 plants/replicates for each cultivar were inspected for first-generation larval nests and/or live larvae. In July and September, all bunches were examined for berries infested with second- and third-generation larval penetration holes, respectively. To determine *L. botrana* infestation levels on Sangiovese and Petit Verdot, two indices were considered, namely, the infestation index (II) and the infestation severity index (SI). The II for each plant was calculated by dividing the number of infested inflorescences or infested bunches by the total number of inflorescences or bunches per plant, respectively [7]. The II represents the proportion of infested units (inflorescences or bunches) and can range from 0 to 1. To assess the severity of infestation, the SI for each plant was calculated as the number of larval nests, larvae, or penetration holes relative to the total number of inspected inflorescences or bunches per plant. The SI quantifies the extent of damage and, since a single bunch may contain multiple nests, larvae, or penetration holes, the SI can exceed 1.

### 2.4. Adult Population Abundance and Dynamics

To study the adult population abundance and dynamics of *L. botrana*, two pheromone traps (TRAPTEST Isagro^®^) per cultivar were placed 1.5 m above the ground, spaced 100 m apart and 27 m from the vineyard edges [40]. The traps were aligned with the two adjacent rows selected for each cultivar (Figure 1). Traps were installed on 1 March 2021, and removed on 28 December of the same year. Pheromone dispensers were replaced every four weeks, while sticky boards were replaced as needed. All traps were checked weekly, and the number of *L. botrana* captures was recorded.

### 2.5. Cultivar Morphological Characterization

The morphological characterization of Petit Verdot and Sangiovese cultivars was carried out in July 2022, after the elongation of the main axis of the shoot and the rachis of the bunch had ceased. The analysis focused on shoot and bunch morphology as well as canopy traits. Each investigated plant represented a replicate.

#### 2.5.1. Shoot Morphology

Ten shoots placed near the trunk of as many vines were selected to determine the number of bunches, the length of the main shoot axis, and the number of laterals present between nodes 2 and 12 starting from the base of the shoot. The main leaves and the laterals on each shoot were collected, labeled with a unique code, and taken to the laboratory for subsequent measurements. These included the length, number of leaves, and blade area of the laterals from nodes 2 to 12, as well as the blade area of the main leaves. The blade areas were measured separately for each shoot using a Li-Cor leaf area meter.

#### 2.5.2. Bunch Morphology

The basal bunch of the ten selected shoots was also collected to determine the rachis length (excluding peduncle) and bunch weight. Bunch compactness was expressed as the ratio of bunch mass to bunch length (g/mm). Bunch density was visually estimated following the Organisation Internationale de la Vigne et du Vin (OIV) code 204 (Organisation Internationale de la Vigne et du Vin 2010), which employs a scale ranging from 1 (very loose bunch: berries clearly separated, many visible pedicels) to 9 (very dense bunch: berries deformed by compression). Berry weight was measured by sampling 50 berries per bunch. The number of berries per bunch was calculated as the ratio of bunch mass to berry mass.

#### 2.5.3. Canopy Traits

Canopy traits of Sangiovese and Petit Verdot cultivars were measured in mid-July, on four plants per cultivar. Canopy height (distance from the lowest and the highest leaf in the selected position) was measured at three positions per vine along the cane, at 0.3 m intervals (from 0.1 to 0.7 m from the trunk). Canopy thickness (the distance between the outermost leaves on each side of the canopy in the selected position) and leaf layer number (LLN) were determined at 18 points per vine: six height levels at 0.2 m intervals (from the supporting cane wire to the top wire catch) and three positions per vine.

### 2.6. Data Elaboration and Statistical Analysis

Data transformation failed to normalize the distributions and homogenize variances of *L. botrana* infestation data. Thus, the non-parametric Mood’s median test (exact method) was used to compare the II and SI between the two grape cultivars. Separate analyses were carried out for each sampling date. The Spearman rank correlation (exact method) was used to investigate whether the number of inflorescences and/or bunches could be a confounding variable for the infestation indices.

For each cultivar, daily trap catches were calculated and a paired-samples sign test (exact method) was performed to investigate the difference in male captures between the two cultivars. Only samples with at least five males per week in each trap were included in the analysis. Weekly trap captures were averaged to describe the male population dynamics on each cultivar.

Data for morphological characterization were tested for homogeneity of variance and analyzed by ANOVA. Treatments were compared using a *t*-test at significance levels of *p* ≤ 0.05 and *p* ≤ 0.01, and the significance levels of the treatments were reported.

All analyses were performed using R (version 4.2.0, Core team 2022).

## 3. Results

### 3.1. Weather Trends

**Figure 2:**
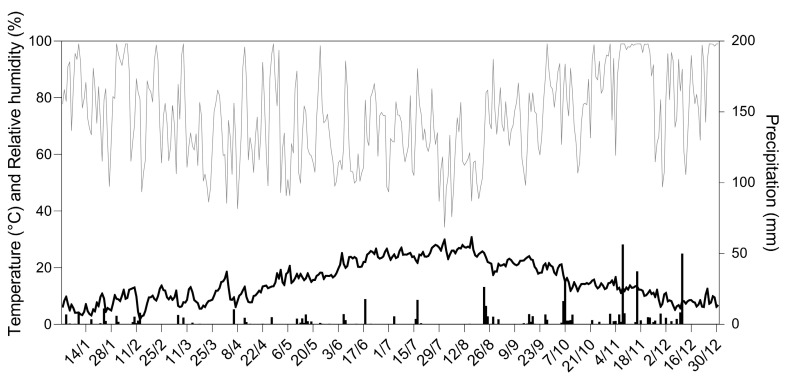
Meteorological trend recorded in Loreto, Ancona province (Italy) in 2021. Bars indicate the daily cumulated precipitation (mm). The bold line represents the daily mean temperature (°C). The gray line represents the daily mean relative humidity (%).

The seasonal weather trend for the year 2021 is presented in Figure 2. Low precipitation levels were observed during spring and summer, with total annual precipitation amounting to 547.40 mm. The average yearly temperature was 15.20 °C. January and July were the coldest and the hottest months, respectively, with an average daily temperature of 6.3 °C in January and 25.2 °C in July.

### 3.2. Infestation Indices

A total of 4716 inflorescences or bunches were visually inspected across the 35 selected plants per cultivar throughout the sampling seasons (Table 1). Of these, 3482 belonged to Petit Verdot (1289 inflorescences in June, 1100 bunches in July, and 1093 in September), while 1234 belonged to the Sangiovese (437 inflorescences in June, 402 bunches in July, and 395 in September). The Spearman rank correlation test showed no significant relationships between the number of inflorescences and/or bunches and the infestation indices.

**Figure 3:**
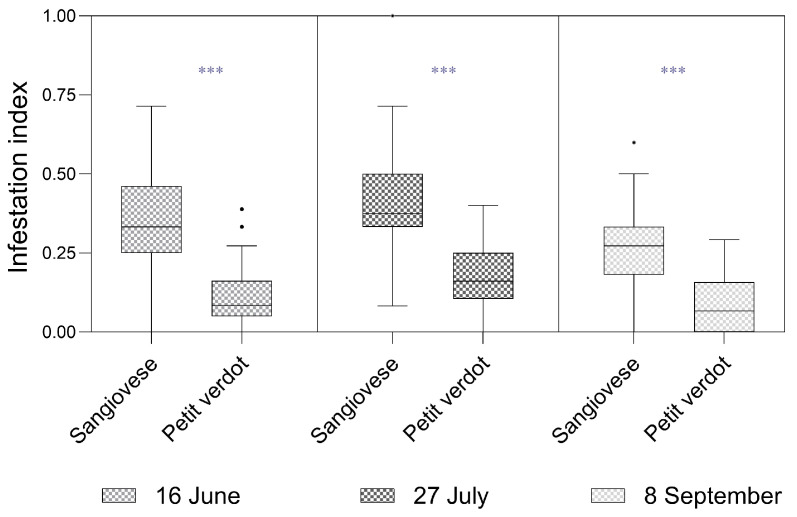
Box plots of the infestation index (II) for Sangiovese and Petit Verdot cultivars measured in June (on inflorescences), July (on bunches), and September (on bunches). The solid line represents the median, while the lower and upper hinges correspond to the first and third quartiles. The whisker extends from the hinge to the smallest/largest value no further than 1.5 times the interquartile range. Points represent outliers. *** Statistical significance at *p*-value < 0.001.

The II was significantly higher in Sangiovese across all three sampling dates (Figure 3). In June, the II (mean ± standard error) was 0.34 ± 0.03 in Sangiovese and 0.12 ± 0.02 in Petit Verdot (Mood’s test, Z = 4.98, *p*-value < 0.001). In July, the II was 0.41 ± 0.03 in Sangiovese and 0.18 ± 0.02 in Petit Verdot, respectively (Mood’s test, Z = 5.93, *p*-value < 0.001). In September, the II was 0.26 ± 0.02 in Sangiovese and 0.09 ± 0.01 in Petit Verdot (Mood’s test, Z = 4.98, *p*-value < 0.001).

**Figure 4:**
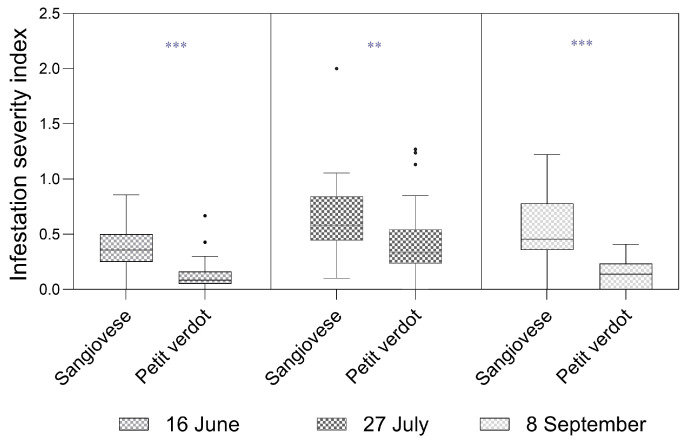
Box plots of the infestation severity index (SI) per plant for Sangiovese and Petit Verdot cultivars measured in June (on inflorescences), July (on bunches), and September (on bunches). The solid line represents the median, while the lower and upper hinges correspond to the first and third quartiles. The whisker extends from the hinge to the smallest/largest value no further than 1.5 times the interquartile range. Points represent outliers. ** and *** Statistical significance at *p*-value < 0.01 and *p*-value < 0.001, respectively.

Similarly, the SI was significantly higher in Sangiovese on all three sampling dates (Figure 4). In June, the SI was 0.39 ± 0.04 in Sangiovese and 0.13 ± 0.02 in Petit Verdot (Mood’s test, Z = 4.98, *p*-value < 0.001). In July, the SI was 0.65 ± 0.06 in Sangiovese and 0.41 ± 0.05 in Petit Verdot, respectively (Mood’s test, Z = 3.32, *p*-value < 0.002). In September, the SI was 0.53 ± 0.05 for Sangiovese and 0.14 ± 0.02 for Petit Verdot (Mood’s test, Z = 5.93, *p*-value < 0.001).

### 3.3. Adult Population Abundance and Dynamics

**Figure 5:**
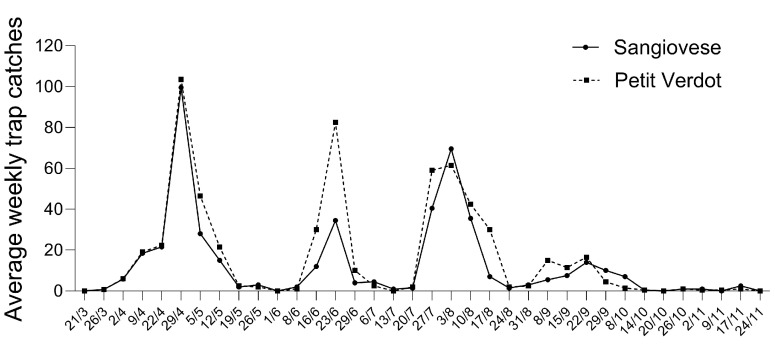
Average weekly adult trap catches of *Lobesia botrana* for each cultivar in Loreto, Ancona province (Italy) in 2021.

The seasonal population dynamic of *L. botrana* across the two cultivars is shown in Figure 5. Adults were captured in traps over approximately seven and a half months, from 21 March to 24 November 2021, with a total of 2.124 individuals recorded. The insect population exhibited four peaks. The average weekly adult population abundance (mean ± SE) across the four traps was relatively high during the first flight period (19.02 ± 4.77) compared to the second (15.33 ± 5.66) and third (12.76 ± 2.59).

The average weekly adult trap count (mean ± SE) for each flight period and cultivar is provided in Table 2. The first flight was the most abundant for Sangiovese, followed by the third and then the second. For Petit Verdot, the highest abundance occurred during the second flight period, followed by the first and the third (Table 2).

**Figure 6:**
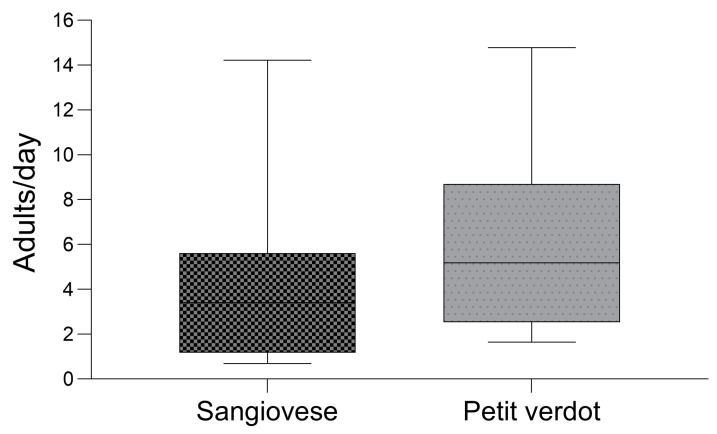
Box plots of the number of males of *Lobesia botrana* sampled in pheromone traps displaced in Sangiovese and Petit Verdot cultivars during the whole flight period (*n* = 35). The solid line represents the median, while the lower and upper hinges correspond to the first and third quartiles. The whisker extends from the hinge to the smallest/largest value no further than 1.5 times the interquartile range.

When considering the daily trap captures across the entire monitoring season, traps placed in Petit Verdot collected a slightly higher number of males (Figure 6). A significant difference in the daily number of males trapped per sampling period between the two grape varieties was observed (*p*-value < 0.01). The number of males (mean ± SE) trapped per trap and per day throughout the sampling season was 6.22 ± 1.21 in Petit Verdot and 4.42 ± 1.18 in Sangiovese. The paired-samples sign test yielded a median difference value of 1.1 males per trap per sampling date (C.I. (0.95%): 0.6~3.1). The effect size, computed by dividing the approximated Z-statistic by the square root of the sample size, was 0.78.

### 3.4. Cultivar Characterization

The characterization of the Petit Verdot and Sangiovese cultivars included data analysis on shoot morphology (Table 3), canopy traits (Table 4), and bunch characteristics (Table 5). Compared to Sangiovese, Petit Verdot exhibited significantly shorter internodes (59 vs. 84 mm), a greater number of laterals per node (0.85 vs. 0.56), and longer lateral shoots per node (112 vs. 52 mm). Accordingly, Petit Verdot had a larger lateral shoot surface area than Sangiovese (201 vs. 123 cm^2^/node). No significant difference was recorded in the leaf blade area on the main shoot axis. The total leaf area per node was still higher in Petit Verdot than in Sangiovese (361 vs. 290 cm^2^/node), though this difference was not statistically significant (Table 3). The combined analysis of internode length and total leaf surface per node can be effectively synthesized by calculating the assigned leaf surface per unit of shoot length. Petit Verdot distributes 360 cm^2^ along only 59 mm of internode length, while Sangiovese distributes 290 cm^2^ along 84 mm. As a result, Petit Verdot has a higher leaf density, with a leaf surface of 6.2 cm^2^ per mm of shoot length, compared to 3.5 cm^2^/mm in Sangiovese. In terms of canopy traits, Petit Verdot exhibited a similar canopy height to Sangiovese (1.24 vs. 1.21 m) but had a thicker canopy (0.43 m vs. 0.23 m) and a greater number of leaf layers (4.8 vs. 2.4). This greater canopy density in Petit Verdot grapevines reduced sun exposure to bunches located in the outer part of the canopy, which were shaded by an average of 1.37 leaf layers, significantly more than the 0.45 leaf layers covering the Sangiovese bunches (Table 4). Petit Verdot bunches were smaller than those of Sangiovese, with a significantly shorter rachis (155 vs. 178 mm), lower mass (101 vs. 213 g), fewer berries (169 vs. 233 mm), and lower berry weight (0.60 vs. 0.91 g at veraison). The bunch density or compactness in Petit Verdot grapevines was markedly lower than in Sangiovese, both visually based on the OIV 204 code (3.2, i.e., loose bunch with berries in loose contact and some visible pedicels, vs. 5.3, i.e., densely packed berries with no visible pedicels, but movable berries) and quantitatively (measured as the ratio of bunch mass to rachis length, Table 5).

## 4. Discussion

Understanding the susceptibility of grape cultivars to *Lobesia botrana* is essential for selecting the most suitable cultivar for a specific area, and for planning and implementing knowledge-based pest management strategies [20,23].

In this study, we evaluated the larval impacts and adult male abundance of *L. botrana* on Petit Verdot and Sangiovese, as well as the morphological characteristics of the investigated cultivars. Our findings revealed that *L. botrana* infestation levels were significantly higher in Sangiovese across all three observed generations. This result aligns with the findings of Baldacchino and Moleas [34], who reported higher infestation levels in Sangiovese compared to other cultivars such as Negroamaro, Uva di Troia, and Trebbiano. The percentages of infested bunches they observed were consistent with the results obtained in our trial for the second and third generations.

Our study reveals that while larval activity was significantly lower in Petit Verdot, the overall abundance of males was higher compared to Sangiovese. Some authors have found a statistically significant correlation between males catches and bunch infestation levels [41,42], but most studies emphasize that male counts from pheromone traps do not reliably correlate with larval density and infestation levels [22,43,44,45]. Our findings suggest potential differences in oviposition success or larval viability across different cultivars and highlight the lack of a clear positive relationship between adult male population abundance and larval infestation levels. In the monitored vineyard, *L. botrana* showed three main peaks, with a fourth, less consistent peak. This pattern has been commonly observed over the last decades in the Marche region, as reported by the monitoring activities conducted by the Phytosanitary Service of the Marche Region (https://meteo.regione.marche.it/Fitopatologia/Parassita/1 (accessed on 29 December 2024)).

Many studies indicate that this pest typically produces three generations per year in central and northern Italy, with a partial fourth generation occasionally occurring in the south [46,47]. The limited number of adult male catches recorded in September might suggest the presence of a partial fourth generation, similar to what was found in northern Italy (Veneto) during the unusually warm summer of 2003 [47]. However, it should be noted that in the study conducted by Marchesini and Dalla Montà [47], fourth-generation catches reached around 300 individuals per trap in early September, whereas this study recorded a peak of just 15 catches per trap. This discrepancy may suggest an alternative explanation: rather than a true fourth generation, the third flight in this vineyard might have displayed a bimodal pattern, with two distinct peaks occurring in early August and late September. This trend has also been reported by Pavan et al. [48] and it is linked to the capacity of *L. botrana* second-generation larvae to develop through either five or six larval instars based on the photoperiod and temperature. The timing and relatively low magnitude of the September peak are unlikely to represent a full fourth generation, as it follows too closely after the third peak, and it involves fewer individuals per trap. Weather trends during the season might provide some insights into the flight patterns of the pest. For first-generation adults to emerge, air temperatures must exceed 10 °C for a sustained period of 10–12 days [49]. Although an initial increase in adult captures began on 26 March, mean daily temperatures dropped below this minimum threshold for about a week starting from 9 April, causing a slower rise in captures at the beginning of the first flight. Additionally, a rainfall event of 10 mm on 6 April likely further inhibited adult flight activity in the days that followed, contributing to the slower increase in adult abundance observed before the first peak. The third flight period, which may have displayed a bimodal pattern, was likely influenced by relatively warm air temperatures observed from late August to mid-October. These conditions likely promoted the presence of two peaks during the third flight. Adult catches persisted into late November with sporadic individuals appearing beyond the third peak. These late-season individuals likely did not survive to reproduce, as temperatures fell at the onset of winter, impeding both survival and reproduction [50].

In the present work, bunch density and bunch compactness (g/mm) were significantly lower in Petit Verdot compared to Sangiovese, both in terms of the ratio between bunch mass and rachis length, as well as according to the OIV 204 code. We hypothesize that the higher infestation levels in Sangiovese are due to a greater bunch density as reported in Fermaud (1998) [51] and Baldacchino and Moleas (2000) [34]. Indeed, bunch compactness plays a crucial role in *L. botrana* infestation levels, likely due to the more favorable microenvironmental conditions it creates, providing suitable sites for oviposition and facilitating the larval viability and development [23,29]. Additionally, compact bunches offer protection from adverse weather and natural predators [51].

Petit Verdot exhibited a significantly greater canopy thickness, leaf layer number, and bunch cover layers of leaves, suggesting that the bunches are less exposed to solar radiation compared to Sangiovese. Based on the results of our study, we hypothesize that the greater leaf coverage of Petit Verdot bunches was not associated with higher *L. botrana* infestation levels and that bunch compactness plays a more important role in larval infestation. Unlike some studies [26,29] in which bunch-zone leaf removal was practiced and lower infestation levels on sun-exposed bunches were reported, no bunch-zone leaf removal was performed in our study. This condition more closely reflects the characteristics of the cultivar when not influenced by any management intervention. We hypothesize that even though the bunches of Sangiovese are not fully exposed to direct sunlight, they still provide suitable conditions for larval settlement and survival. The presence of *L. botrana* may have been further facilitated by the high bunch compactness exhibited by this cultivar, which has been found to favor the presence of the pest, particularly during carpophagous generations [22,34,52]. Despite the higher male captures observed in Petit Verdot, it cannot be concluded from this study that females are able to lay more eggs on this cultivar [53], nor can it be inferred that larval survival is compromised due to the looser cluster structure of this cultivar. Further studies are needed to gain a deeper understanding of the role of cultivar-specific traits in oviposition success and larval development by *L. botrana*.

## 5. Conclusions

In conclusion, this study provides useful insight into the population abundance and impacts of *L. botrana* on Petit Verdot and Sangiovese, providing elements for evaluating how cultivar-specific traits may influence pest infestation levels. These findings represent the first detailed examination for Petit Verdot, which is gaining significant attention in countries including Spain, the United States, Australia, France, Chile, South Africa, Argentina, and Italy. The study offers valuable information for more informed cultivar selection, and for optimizing pest monitoring and control strategies. For example, monitoring efforts could be prioritized for more susceptible cultivars, where the likelihood of finding larvae is higher. Additionally, action thresholds could be refined based on the specific susceptibility of each cultivar, enabling more targeted and effective pest management.

## Figures and Tables

**Table 1:** Total number of inflorescences or bunches inspected and infested by *Lobesia botrana* in the three sampling periods (1st generation in June inflorescences, 2nd generation in July bunches, and 3rd generation in September bunches) for Sangiovese and Petit Verdot cultivars.

Cultivar	1st Generation		2nd Generation		3rd Generation	
	Total #	Infested #	Total #	Infested #	Total #	Infested #
Sangiovese	437	150	402	162	395	102
Petit Verdot	1289	140	1100	179	1093	103

“#” refers to number

**Table 2:** Average weekly adult trap catches (±SE) for each flight period of *Lobesia botrana* on Sangiovese and Petit Verdot cultivars.

Cultivar	1st Flight Period	2nd Flight Period	3rd Flight Period
Sangiovese	17.6 (±6.7)	9.7 (±4.9)	11.5 (±3.4)
Petit Verdot	20.4 (±6.9)	21.0 (±10.2)	14.0 (±3.9)

**Table 3:** Shoot traits of the Sangiovese and Petit Verdot cultivars measured in mid-July between internode 2 and internode 12 from the shoot base. Mean (±SE) of 10 shoots near the trunk of the grapevines.

Cultivar	Internode Length (mm)	Laterals (#/Node)	Lateral Length (mm/Node)	Lateral Blade Area (cm^2^/Node)	Main Leaf Area (cm^2^/Node)	Total Leaf Area (cm^2^/Node)
Sangiovese	83.50 (±3.44)	0.56 (±0.06)	52.35 (±9.28)	122.83 (±18.46)	167.10 (±7.20)	289.93 (±22.05)
Petit Verdot	58.60 (±2.59)	0.85 (±0.03)	112.30 (±14.98)	200.84 (±27.87)	159.65 (±5.28)	360.49 (±30.05)
F Sign.	**	**	**	*	n.s.	n.s.

* Significant at *p*-value < 0.05. ** Significant at *p*-value < 0.01. n.s. Not statistically significant. “#” refers to number

**Table 4:** Canopy traits (height, thickness, and leaf layer number) and bunch cover of Sangiovese and Petit Verdot grapevines measured in mid-July. Canopy height was determined in three positions per vine along the cane at 0.3 m distance (from 0.1 to 0.7 m from the trunk of the grapevine). Thickness and leaf layer number (LLN) were determined at 18 points per vine: 6 height levels at 0.2 m distance (from supporting cane wire to the last couple of catch wires) and 3 positions per vine along the cane at 0.3 m distance (from 0.1 to 0.7 m from the trunk of the grapevine). Results are reported as mean (±SE).

Cultivar	Canopy Height (m)	Canopy Thickness (m)	Leaf Layer Number (#)	Bunch Cover Layers of Leaves (#)
Sangiovese	1.21 (±0.03)	0.23 (±0.01)	2.43 (±0.13)	0.45 (±0.06)
Petit Verdot	1.24 (±0.06)	0.43 (±0.02)	4.83 (±0.21)	1.37 (±0.21)
F Sign.	n.s.	**	**	**

** Significant at *p*-value < 0.01. n.s. Not statistically significant. “#” refers to number

**Table 5:** Bunch morphology of Sangiovese and Petit Verdot grapevines measured in mid-July. The density or compactness of the bunch was assessed both visually according to the Organisation Internationale de la Vigne et du Vin 204 code with a classification scale ranging from 1 (very loose bunch: berries clearly separated, many visible pedicels) to 9 (very dense bunch: berries deformed by compression) and as the ratio of bunch mass and rachis length (g/mm). Results are reported as mean (±SE).

Cultivar	Rachis Length (mm)	Bunch Mass (g)	Berries per Bunch (#)	Berry Weight (g)	Bunch Density (OIV 204)	Bunch Compactness (g/mm)
Sangiovese	178.40 (±7.61)	212.70 (±15.39)	233.97 (±12.87)	0.91 (±0.04)	5.30 (±0.21)	1.20 (±0.08)
Petit Verdot	154.90 (±7.98)	101.40 (±11.17)	169.92 (±15.42)	0.60 (±0.02)	3.22 (±0.20)	0.67 (±0.07)
F Sign.	*	**	**	**	**	**

* Significant at *p*-value < 0.05. ** Significant at *p*-value < 0.01. “#” refers to number

## Data Availability

Data are available upon reasonable request.

## Abbreviations

The following abbreviations are used in this manuscript:

IPM	integrated pest management
II	infestation index
SI	infestation severity index
OIV	Organisation Internationale de la Vigne et du Vin
LLN	leaf layer number
